# Noninvasive Measurement of Conductivity Anisotropy at Larmor Frequency Using MRI

**DOI:** 10.1155/2013/421619

**Published:** 2013-03-11

**Authors:** Joonsung Lee, Yizhuang Song, Narae Choi, Sungmin Cho, Jin Keun Seo, Dong-Hyun Kim

**Affiliations:** ^1^Department of Electrical and Electronic Engineering, Yonsei University, Seoul 120-749, Republic of Korea; ^2^Department of Computational Science & Engineering, Yonsei University, Seoul 120-749, Republic of Korea

## Abstract

Anisotropic electrical properties can be found in biological tissues such as muscles and nerves. Conductivity tensor is a simplified model to express the effective electrical anisotropic information and depends on the imaging resolution. The determination of the conductivity tensor should be based on Ohm's law. In other words, the measurement of partial information of current density and the electric fields should be made. Since the direct measurements of the electric field and the current density are difficult, we use MRI to measure their partial information such as B1 map; it measures circulating current density and circulating electric field. In this work, the ratio of the two circulating fields, termed circulating admittivity, is proposed as measures of the conductivity anisotropy at Larmor frequency. Given eigenvectors of the conductivity tensor, quantitative measurement of the eigenvalues can be achieved from circulating admittivity for special tissue models. Without eigenvectors, qualitative information of anisotropy still can be acquired from circulating admittivity. The limitation of the circulating admittivity is that at least two components of the magnetic fields should be measured to capture anisotropic information.

## 1. Introduction

 Noninvasive measurement of electrical properties for biological tissues can be useful in EEG/MEG and electromagnetic source imaging [1] and in providing diagnostics information about the physiological and pathological states of the tissues [2–5]. For isotropic conductivity, many approaches have been developed to measure the conductivity at low frequencies and at Larmor frequencies [6]. At low frequencies below 1 kHz, Magnetic Resonance Electrical Impedance Tomography (MREIT) [7] can probe the conductivity distribution. At Larmor frequencies of about 100 MHz, Magnetic Resonance Electrical Property Tomography (MREPT) [8, 9] measures both electric conductivity and permittivity distributions using measurements of positively rotating magnetic fields generated by transmit RF coil, *B*1+ maps, from MRI.

Microscopically, the conductivity of the biological tissues could be isotropic. However, depending on the imaging resolution, the conductivity of an imaging voxel can be anisotropic. Macroscopically, in other words, if several tissues with different electrical properties are combined in the imaging voxel, the conductivity of the imaging voxel differs when measured in different directions so that it becomes anisotropic. Especially in biological tissues, anisotropic electrical conductivity can be found in muscles and nerves [2–5]. The conductivity tensor is a simplified model with three eigenvectors and three eigenvalues which can include these anisotropic cases.

The eigenvectors of the conductivity tensor at low frequencies (<1 kHz) can be inferred from a prior knowledge of the object or the diffusion tensor imaging [1, 10]. However, at Larmor frequency of about 100 MHz, there have been a few studies on measuring three eigenvectors and eigenvalues of the conductivity tensor. Recently, Katscher et al. [11] proposed a way to estimate partial information of conductivity anisotropy especially in the special case where two minimum eigenvalues are almost equal to zero. In this work, based on Katscher's approach [11], we generalized and considered more possible cases. Based on numerical phantom simulations and phantom experiments, the performance was evaluated and the limitations and future directions were proposed.

## 2. Materials and Methods

### 2.1. Admittivity Tensor Model

Admittivity tensor denoted by **κ**(**r**) = **σ**(**r**) + *iω* **ϵ**(**r**) is a simplified model for the electrical anisotropic information at the angular frequency *ω*, where **σ**(**r**) and **ϵ**(**r**) are conductivity and permittivity tensors, respectively.

The admittivity tensor can be represented with six parameters:

(1) κ(r)=(κxx(r)κxy(r)κxz(r)κxy(r)κyy(r)κyz(r)κxz(r)κyz(r)κzz(r)).

Expressing its eigenvectors **v**_1_, **v**_2_, **v**_3_ (unit vectors) and its corresponding eigenvalues *κ*_1_, *κ*_2_, *κ*_3_, the admittivity tensor can be also expressed as

(2) κ=(v1v2v3)(κ1κ2κ3)(v1Tv2Tv3T).

We should note that three equations of Ohm's law **J** = **κ** **E** alone are insufficient to identify six unknown components of **κ**. However, the eigenvectors of the conductivity tensor **κ** could be estimated from prior knowledge of the object or can be determined by measuring the diffusion tensors [1] using MRI. Under the assumption that the eigenvectors of the admittivity tensor are known *a priori*, using the eigenvectors **v**_1_, **v**_2_, **v**_3_ of the matrix **κ**, the conductivity tensor can be decomposed as

(3) ∑j=13Jj(r)vj=J(r)=κ(r)E(r)=κ(r)[∑j=13Ej(r)vj]=∑j=13κjEj(r)vj,

where *J*_*j*_ = **J** · **v**_*j*_ and *E*_*j*_ = **E** · **v**_*j*_. In other words, *J*_*j*_(**r**) = *κ*_*j*_ *E*_*j*_(**r**).

### 2.2. Reconstruction of Admittivity Anisotropy Using Measured Magnetic Fields: Circulating Admittivity

Assume that the three eigenvectors are known. The effective admittivity, **κ** = **σ** + *iω* **ϵ**, in a voxel can be determined from Ohm's law as follows:

(4) κj∫VoxelE(r)·vj dr=∫VoxelJ(r)·vj dr.

However in MRI, **E** and **J** are hard to measure. Instead, partial knowledge of the magnetic fields **H** can be acquired from *B*1 mapping techniques [12–15]. Katscher et al. [11] extended the direct inversion method in MREPT [9] and proposed a way to estimate anisotropy of **κ** using the relationship between circulating currents and circulating electric fields over a surface, which can be estimated from the measured magnetic fields.

From time-harmonic Maxwell Equations, the relationship among the current density, electric fields, and the magnetic fields can be expressed as

(5) J(r)=∇×H(r),  ∮∂A(n)E·dl=−iωμ∫A(n)H·ds,

where *A*(**n**) is a surface whose normal vector is **n**.

Based on the work by Katscher et al. [11], we define the circulating admittivity, $\tilde{\kappa }$, as the ratio of the circulating currents and the circulating electric fields over the surface *A*(**n**) with rotating the normal vector **n**:

(6) κ~(A(n)):=∮∂A(n) J·dl∮∂A(n) E·dl=−1iωμ∮∂A(n)∇×H·dl∫A(n)H·ds=1iωμ∫A(n)∇2H·ds∫A(n)H·ds,

where **s** is parallel to the normal vector **n**. In (6), the relationship between the curl integral of ∇  × **H** and the surface integral of ∇^2^ **H** hold for homogeneous region of admittivity and can generate artifacts at tissue boundaries [16].

In this work, we investigated the relationship between the circulating admittivity and the admittivity tensor for simple cases. First, if the admittivity tensor is isotropic, the circulating admittivity is also isotropic and equal to the isotropic admittivity. Second, if two eigenvalues of the admittivity tensor are the same, *κ*_2_ = *κ*_3_, the eigenvalue can be determined from the circulating admittivity over a surface with a normal vector, **v**_1_, perpendicular to the eigenvectors corresponding to the two eigenvalues, *κ*_2_, *κ*_3_. That is

(7) κ~(A(v1)):=∮∂A(v1)J·dl∮∂A(v1)E·dl=∮∂A(v1)(κ2E2v2+κ3E3v3)·dl∮∂A(v1)(E2v2+E3v3)·dl=κ2,

since the normal vector **v**_1_ is perpendicular to the vector *d* **l** over a line ∂*A*(**v**_1_) and the two eigenvalues are the same *κ*_2_ = *κ*_3_.

In addition, as it is considered in [9], if two smaller eigenvalues of the admittivity tensor, *κ*_2_, *κ*_3_, are equal to zero, the largest eigenvalue, *κ*_1_, can be determined from the circulating admittivity directly:

(8) κ~(A(n))=∮∂A(n) κj1E1v1·dl∮∂A(n) E1v1·dl=κ1∮∂A(n) E1v1·dl∮∂A(n) E1v1·dl=κ1.

As a combination of last two cases, if the largest eigenvalue is much bigger than the two smaller eigenvalues of the admittivity tensor and the two smaller eigenvalues are the same, by measuring the circulating admittivity for several directions of the normal vector, all three eigenvalues could be estimated.

### 2.3. Unknown Directions of Eigenvectors: Effective Admittivity Map (EAM) and Circulating Admittivity Map (CAM)

 In the previous section, we determined the admittivity tensor under the assumption that the eigenvectors of the admittivity tensor were known *a priori*. Even without the prior knowledge of the eigenvectors, we can still provide a qualitative measurement of anisotropy by computing the dependency on the normal vector, **n**, in (6). For a qualitative measurement of the anisotropy, we define the effective admittivity map, $\bar{\kappa }\left(r,n\right)$, and the circulating admittivity map, $\hat{\kappa }(r,n)$, that describe the distributions of effective admittivity and circulating admittivity over the normal vector, respectively:

(9) $$\begin{array}{c} \bar{\kappa }\left(r,n\right):=\frac{\int J\left(r\right)\cdot n\;dr}{\int E\left(r\right)\cdot n\;dr},\\ \hat{\kappa }\left(r,n\right):=\frac{1}{i\omega \mu }\frac{\int \nabla^{2}H\left(r\right)\cdot n\;dr}{\int H\left(r\right)\cdot n\;dr}. \end{array}$$

As shown in Figure 7, in a later section, the effective admittivity map (EAM) and the circulating admittivity map (CAM) can be drawn with the use of two angles,   *θ*_*xy*_ and *θ*_*xz*_ which describe the direction of the normal vector. That is, the normal vector was initially located at the positive *z*-axis, was rotated along *y*-axis by *θ*_*xz*_, and then was rotated along *z*-axis by *θ*_*xy*_. 

### 2.4. Numerical Simulation: Numerical Phantom Model with Anisotropic Effective Admittivity

For numerical evaluation, a numerical phantom with anisotropic effective admittivity can be generated using periodic binary medium. According to homogenization theory, anisotropy can be derived from pointwise admittivity, *κ*(**r**), that is distributed periodically with respect to the *y*-variable:

(10) κ(r):={κ1=σ1+iωϵ1if    0≤Ny−[Ny]<c,κ2=σ2+iωϵ2if    c≤Ny−[Ny]<1,

where 0 < *c* < 1 is a constant depending on the binary medium, *N* is a large positive integer, and [*Ny*] is the largest integer not greater than *Ny*.

**Figure 1:**
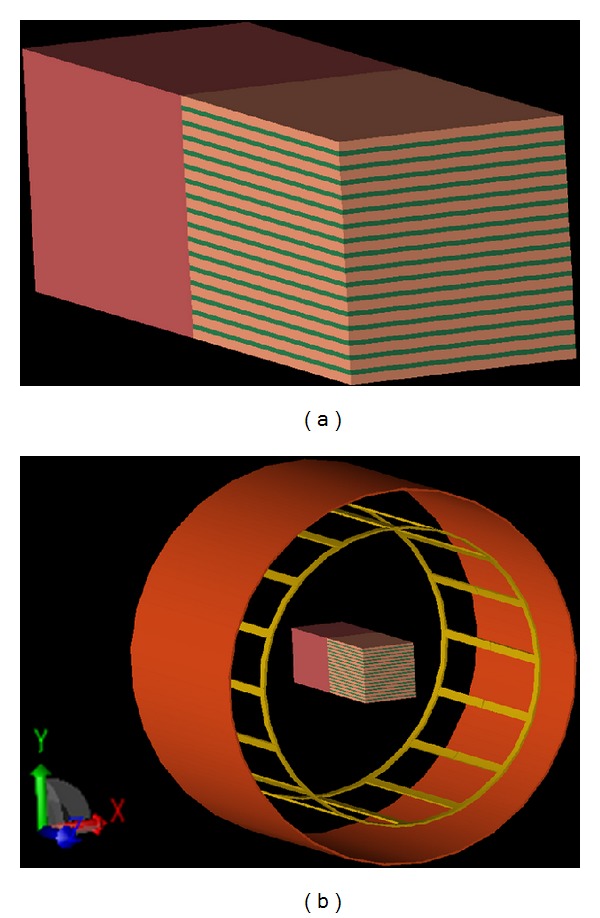
(a) Numerical phantom with anisotropic effective admittivity by stacking periodic binary medium, (b) placement of the imaging object inside the RF coil.

In this numerical experiment the imaging subject is the box *Ω* : = [−50,50]×[−50,50]×[−80,80] mm^3^. We divided the domain *Ω* into two subdomains *Ω* ^0^ : = {**r** ∈ *Ω* : *z* < 0} and *Ω* ^*a*^ : = {**r** ∈ *Ω* : *z* > 0}. In *Ω* ^0^ the admittivity is homogeneous with the value *κ* = 1 + *iωϵ*, where the permittivity *ϵ* = 80*ϵ*_0_ with *ϵ*_0_ the permittivity in the free space. In *Ω* ^*a*^ 33 layers were stacked alternatively with the thickness of 2 mm, the admittivity value *κ*_1_ = 5 + *iωϵ* and the thickness of 1 mm, and the admittivity value *κ*_2_ = 0.3 + *iωϵ* in (10) with setting *c* to be 2/3.  Figure 1(a) shows the construction of the imaging object.

Driven by a birdcage coil at 3T (*ω* = 128 MHz) as shown in Figure 1(b), the electric fields, the magnetic fields, and the current densities in microscale were calculated using finite-difference time domain (FDTD) numerical simulations using REMCOM (REMCOM, State College, PA) with the resolution of 1 mm × 1 mm × 1 mm. Then, to determine the effective admittivity in macroscale, that is, the ratio of the ensemble mean current density to the ensemble mean electrical field, three-dimensional Gaussian filter with the size of 17 × 17 × 17 mm^3^ and the standard deviation of 2.0 mm, which increases the effective voxel size from (1 mm)^3^ to (5 mm)^3^, was applied to the simulated fields. 

### 2.5. MRI Experiments

**Figure 2:**
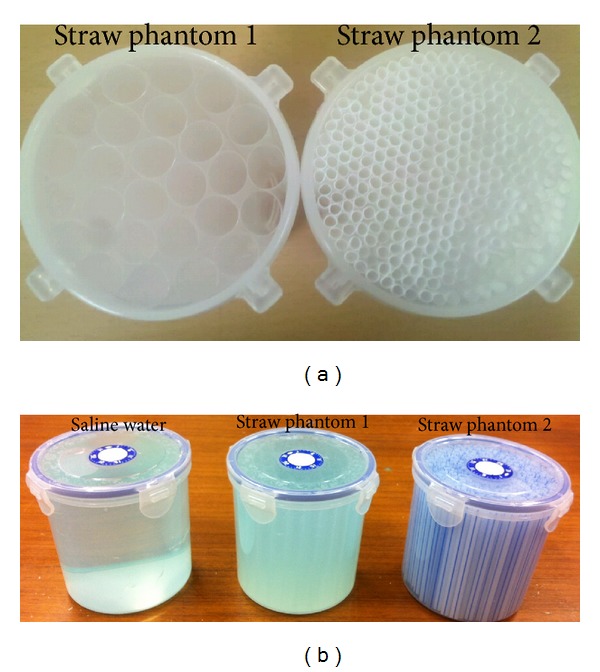
(a) Two straw phantoms before filling saline water, (b) three phantoms filled with saline water.

Two phantoms with anisotropic admittivity were generated using straws as shown in Figure 2. As a comparison, one water phantom with isotropic admittivity was made without straws. Three phantoms were cylindrical with the height of 120 mm and the radius of 50 mm. The diameters of the straws are 12 mm for straw phantom  1 and 6 mm for straw phantom  2. All three phantoms were filled with the saline water of 0.35 M NaCl concentration as shown in Figure 2.

Using a single-channel transreceive head coil, MR images were measured. The phantoms were located at the isocenter of the coil with the straw orientation of left-right. Only the phase of *H* ^+^ was measured and the circulating conductivity was determined by the phase-based approximation in MREPT [17]. Three-dimensional balanced steady-state free procession (bSSFP) was acquired with resolution of 3 mm × 3 mm × 3 mm, field of view (FOV) of 384 mm × 192 mm × 144 mm, and image size of 128 × 64 × 48. The other imaging parameters were the flip angle of 30 degrees, TE of 1.8 ms, TR of 3.6 ms, and the scan time of 5 minutes with 27 averages. All measurements were performed on a 3T Siemens Tim Trio scanner. The phase of *H* ^+^ was estimated as the half of the measured phase of the image [17].

## 3. Results

### 3.1. The Effective Admittivity of the Numerical Phantom

**Figure 3:**
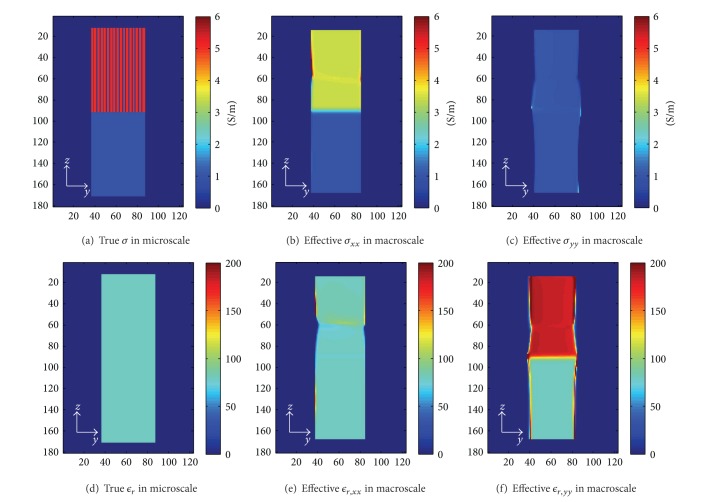
(a) and (d) depict the true conductivity and the relative permittivity values in the microscopic scale. (b)–(f) illustrate the true effective *σ*_*xx*_, *σ*_*yy*_, *ϵ*_*xx*_, and *ϵ*_*yy*,_ respectively.

Based on (4), by dividing the filtered current densities and filtered electric fields, the effective anisotropic material can be acquired. Figures 3(a) and 3(d) illustrate the conductivity and the relative permittivity in microscale of 1 mm resolution in the slice at {*x* = 10 mm} that we set in this simulation, respectively. The effective conductivities in macroscale *σ*_*xx*_, *σ*_*yy*_ are shown in Figures 3(b) and 3(c) and the effective relative permittivities are shown in Figures 3(e) and 3(f). For the subdomain of homogeneous tissue, *Ω* ^0^, the conductivity and relative permittivity are constant and the same microscopically and macroscopically. The effective conductivity and relative permittivity are almost constant for the subdomain of the alternating layers of tissues, *Ω* ^*a*^ except some distortions, we think, due to simulation errors. Since in this experiment *κ*_*zz*_ is the same as *κ*_*xx*_, *κ*_*zz*_ was not shown here.

### 3.2. Observation of Anisotropy Using Circulating Admittivity (6)

In MRI, only partial information of the magnetic fields can be measured. Using conventional single-transmit channel MR scanner, the circularly polarized component of the magnetic fields, *H* ^+^ : = (*H*_*x*_ + *iH*_*y*_)/2, can be measured but the other two components, *H* ^−^ : = (*H*_*x*_ − *iH*_*y*_)/2, *H*_*z*_, are hard to measure. Using a specialized scanner, parallel transmit system, the anticircularly polarized component could be measured [15]. Here, we considered two cases: (1) using *H* ^+^ and *H* ^−^, (2) using only *H* ^+^. For the computation of the circulating admittivity using a partial information of the magnetic fields, the unmeasured magnetic fields were assumed to be zero.

**Figure 4:**
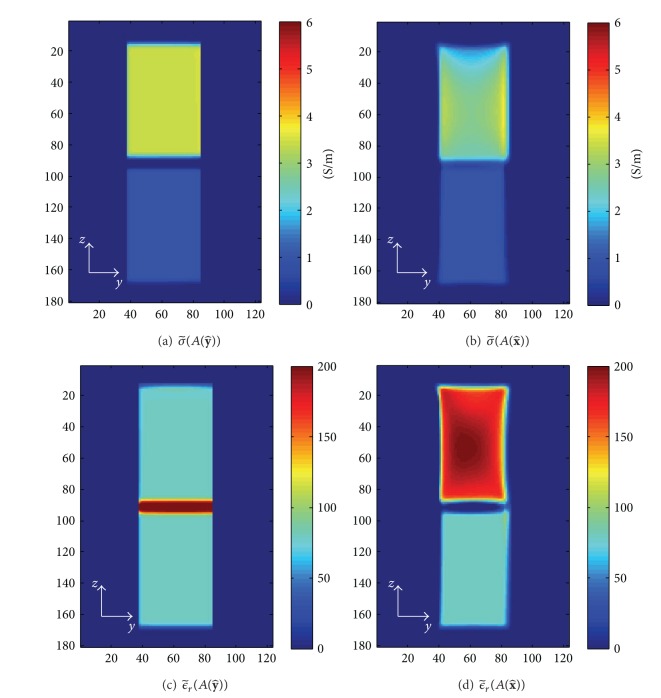
Circulating conductivity and relative permittivity derived from *H* ^+^ and *H* ^−^: (a) $\tilde{\sigma }(A(\hat{y}))$, (b) $\tilde{\sigma }(A(\hat{x}))$, (c) ${\tilde{\epsilon }}_{r}(A(\hat{y}))$, and (d) $\;{\tilde{\epsilon }}_{r}(A(\hat{x}))$.

Figure 4 illustrates the values of circulating conductivity and the relative permittivity, $\tilde{\sigma }(A(n)):=ℜ(\tilde{\kappa }(A(n))$ and ${\tilde{\epsilon }}_{r}(A(n)):=ℑ(\tilde{\kappa }(A(n)))/\omega \epsilon_{0}$, with *H* ^+^ and *H* ^−^ at the slice, *x* = 10 mm. The surface *A*(**n**) for the integration (6) was chosen as a plane with the size of 5 × 5 × 1 pixels^3^ and the normal vector, **n**, of $\hat{x}$, $\hat{y}$. In this case, the two components of the magnetic fields, *H* ^+^ and *H* ^−^, were assumed to be known and used to reconstruct the circulating admittivity. As derived in (7), the circulating admittivity with the normal vector of $\hat{y}$, which is perpendicular on the two eigenvectors of the effective admittivity tensor with the same eigenvalues, is close to the effective admittivity in $\hat{x}$ direction except at the tissue boundaries. For the normal vector of $\hat{x}$, the circulating admittivity is a weighted average of the effective admittivities, *κ*_*xx*_, *κ*_*yy*_, *κ*_*zz*_. Thus, the circulating conductivity with the normal vector of $\hat{x}$ is bigger than the effective conductivity corresponding to the smallest eigenvalue of the effective admittivity tensor, *σ*_*yy*_, shown in Figure 3(c).

**Figure 5:**
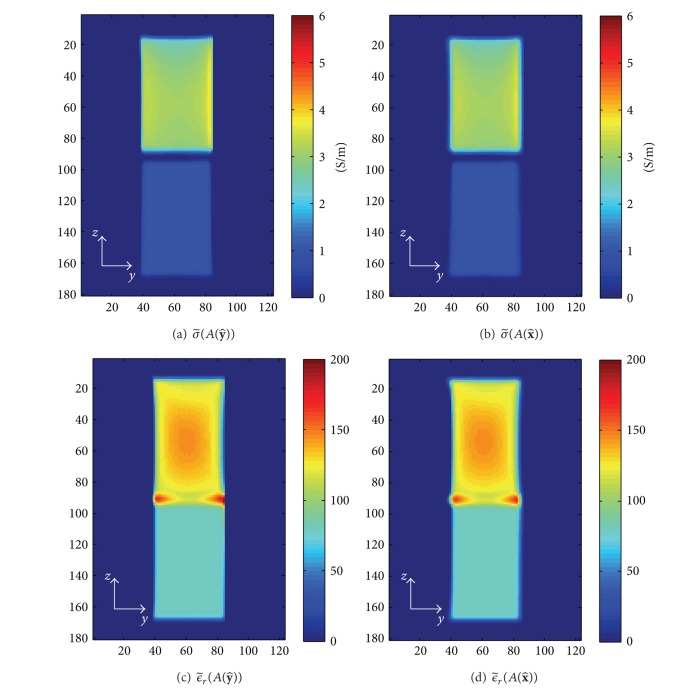
Circulating conductivity and relative permittivity derived from only *H* ^+^: (a) $\tilde{\sigma }(A(\hat{y}))$, (b) $\tilde{\sigma }(A(\hat{x}))$, (c) ${\tilde{\epsilon }}_{r}(A(\hat{y}))$, and (d) ${\tilde{\epsilon }}_{r}(A(\hat{x}))$.

**Figure 6:**
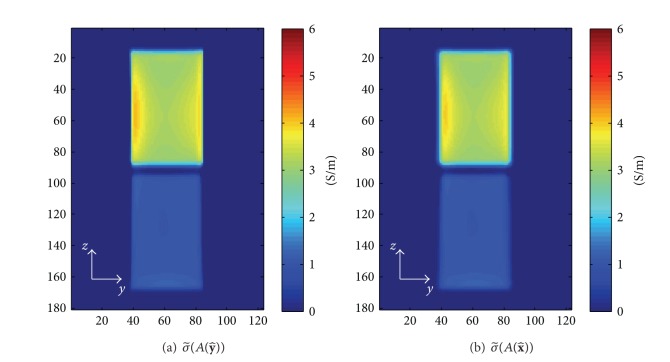
Circulating conductivity derived with only phase of *H* ^+^, (a) $\tilde{\sigma }(A(\hat{y}))$, (b) $\tilde{\sigma }\left(A\left(\hat{x}\right)\right)$.

 However, as shown in Figures 5 and 6, if only one component, *H* ^+^, is available, the dependency on the normal vector was lost in the circulating admittivity.

**Figure 7:**
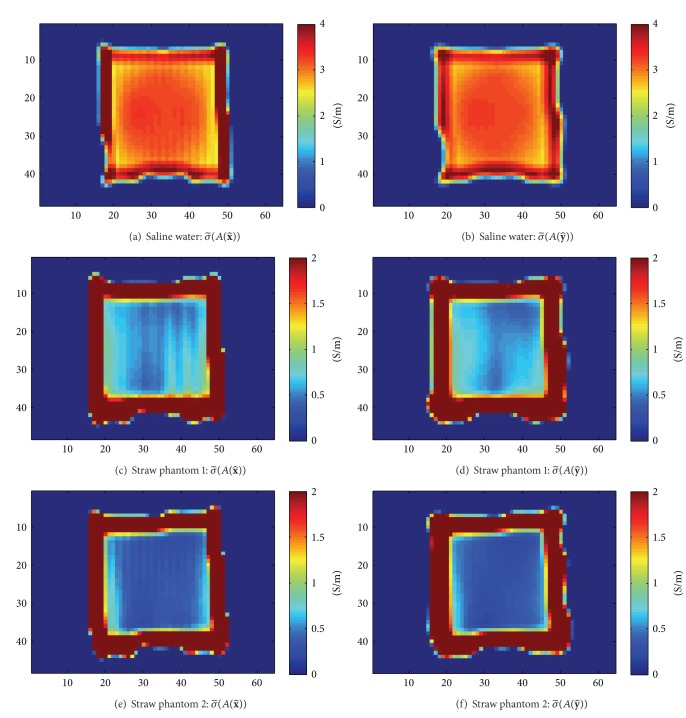
Experiment results: circulating conductivity of phantoms with measured *H* ^+^, Coronal Slice, (a) $\tilde{\sigma }(A(\hat{x}))$ of saline water, (b) $\tilde{\sigma }(A(\hat{y}))$ of saline water, (c) $\tilde{\sigma }(A(\hat{x}))$ of straw phantom 1, (d) $\tilde{\sigma }(A(\hat{y}))$ of straw phantom 1, (e) $\tilde{\sigma }(A(\hat{x}))$ of straw phantom 2, (f) $\tilde{\sigma }(A(\hat{y}))$ of straw phantom 2.

 The circulating conductivities were determined from phantom experiments in which only the phase of *H* ^+^ is measurable. As shown in Figure 7, the dependency on the normal vector was also lost in the experimental results. However, the decrease of the conductivity due to the plastic straws was observed.

### 3.3. Distribution of the Admittivity: Circulating Admittivity Map (CAM)

**Figure 8:**
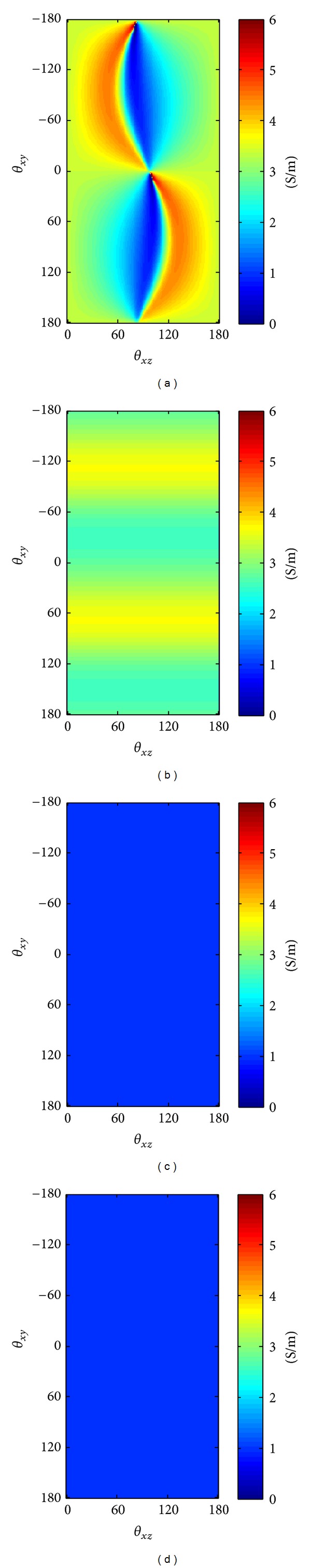
Effective conductivity maps and circulating conductivity maps of one anisotropic voxel and one isotropic voxel: (a) effective conductivity map of anisotropic voxel, *x* = 10 mm, *y* = 0 mm, *z* = 40 mm, (b) circulating conductivity map of anisotropic voxel, *x* = 10 mm, *y* = 0 mm, *z* = 40 mm, (c) effective conductivity map of isotropic voxel, *x* = 10 mm, *y* = 0 mm, *z* = −40 mm, (d) circulating conductivity map of isotropic voxel, *x* = 10 mm, *y* = 0 mm, *z* = −40 mm.

 Using simulated magnetic fields, *H* ^+^ and *H* ^−^, the circulating admittivity map (CAM) was computed for the numerical phantom. As a comparison, the effective conductivity map was also computed using simulated current density and electric fields. The *θ*_*xy*_ and *θ*_*xz*_, which determined the direction of the normal vector **n**, varied from −180° ~  180° and 0° ~ 180° by one degree.  Figure 8 shows the effective conductivity maps, the real part of the EAM, and the circulating conductivity maps, the real part of the CAM, of one anisotropic voxel located at *x* = 10 mm, *y* = 0 mm, and *z* = 40 mm and one isotropic voxel located at *x* = 10 mm, *y* = 0 mm, and *z* = −40 mm. 

 For the isotropic voxel, the values of the effective conductivity map and circulating conductivity map were equal to the conductivity of the tissue. For the anisotropic voxel, the circulating conductivity map is also uniform along the direction *θ*_*xz*_ since only *H* ^+^ and *H* ^−^ were used. In both effective conductivity maps and circulating conductivity maps, the direction that maximizes or minimizes the conductivity value does not match with any eigenvector of the admittivity tensor. Thus, with CAM alone, the eigenvectors of the admittivity tensor may be hard to determine and thus a quantitative measurement of the eigenvalues may be hard.

**Figure 9:**
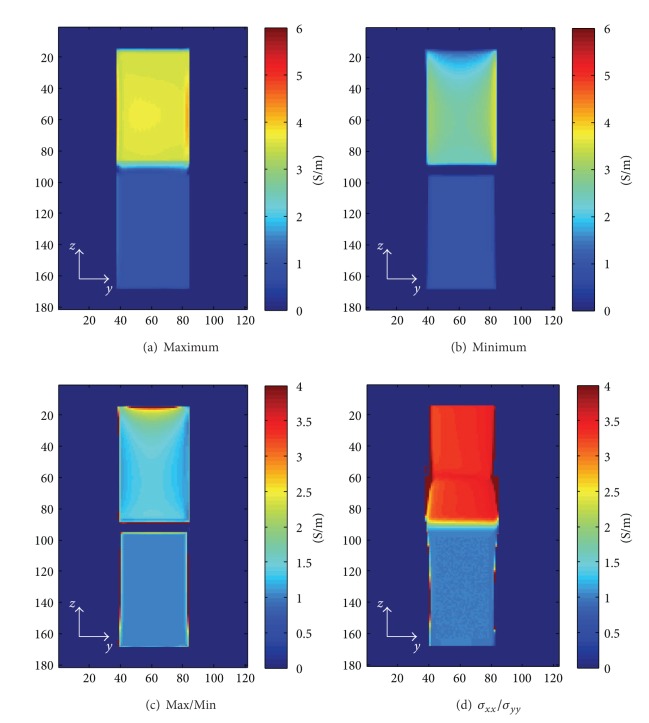
The distribution of the circulating conductivity: (a) the maximum values for the circulating conductivity maps, (b) the minimum values for the circulating conductivity maps, (c) the ratio of the maximum to the minimum, (d) the ratio of two eigenvalues in the conductivity tensor.

For a qualitative analysis, at each voxel, the maximum value, the minimum value, and the ratio of the maximum to the minimum of the circulating conductivity maps were computed with *H* ^+^ and *H* ^−^. As shown in Figure 9, in this case, the maximum values were almost constant over anisotropic tissues, but the minimum values were not constant. The ratio of the maximum to minimum, which could be used as a qualitative measurement of the anisotropy, was not constant over anisotropic tissues and was smaller than the ratio of the maximum eigenvalue, *σ*_*xx*_, to the minimum eigenvalue, *σ*_*yy*_, of the conductivity tensor; that is, using CAM, the contrast between isotropic and anisotropic tissues was reduced. However, CAM still can separate anisotropic tissues from isotropic tissues without knowing the eigenvectors of the admittivity tensor.

## 4. Discussion

Conductivity tensor is a simplified anisotropy model. Given three eigenvectors, the tensor can be estimated if the electric current densities and electric fields can be measured. In MRI, however, electric fields are hard to be measure without knowing or estimating the conductivity and permittivity of tissues. In this work, using MREPT formulae, the circulating admittivity is proposed as a measure to analyze the anisotropy of the tissues. Circulating admittivity was defined as the ratio of circulating current densities to the circulating electric fields, which can be determined from the magnetic fields. We did not fully investigate, but we derived the relationship between the admittivity tensor for special cases. Using numerical phantom simulations, we verified the relationship for the first two cases: (1) isotropic tissues and (2) two eigenvalues of the admittivity tensor are the same. As a future work, more realistic cases would be considered.

In this work, to deal with unknown eigenvectors, the circulating admittivity map (CAM) was proposed as a qualitative measure. The ratio of the maximum to the minimum conductivity was reduced but still anisotropic tissues can be separated from isotropic tissues.

**Figure 10:**
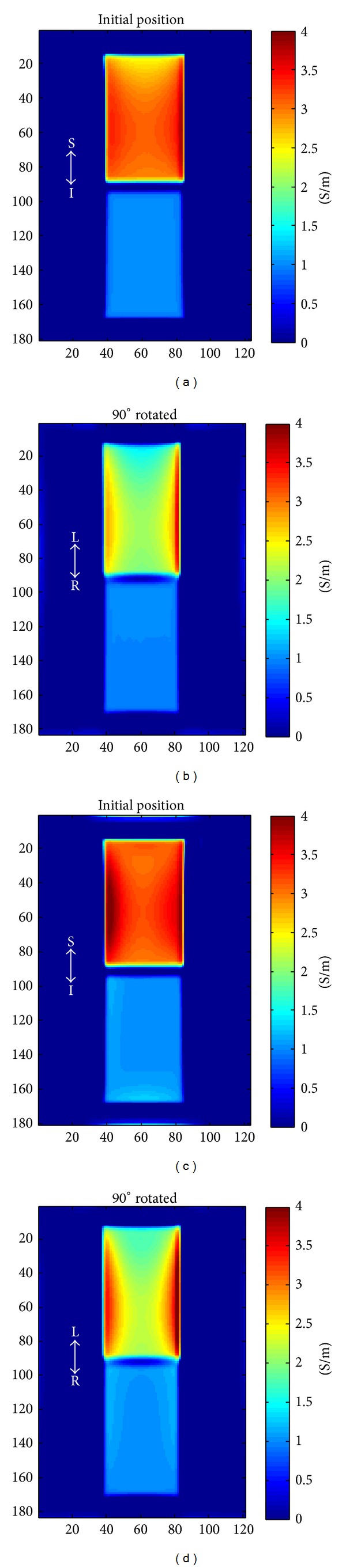
Simulation results: circulating conductivity of simulated phantoms with measured *H* ^+^, coronal slice: (a) initial position using both magnitude and phase of *H* ^+^, (b) 90° rotated using both magnitude and phase of *H* ^+^, (c) initial position using only the phase of *H* ^+^, and (d) 90° rotated using only the phase of *H* ^+^.

**Figure 11:**
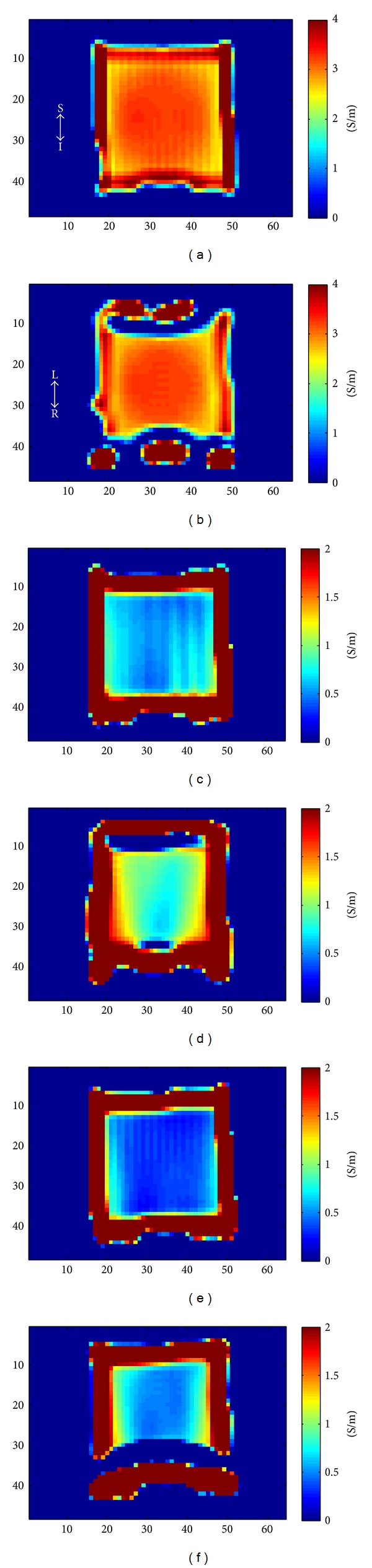
Experiment results: circulating conductivity of phantoms with measured *H* ^+^, coronal slice, (a) saline water: initial position, (b) saline water: 90° rotated, (c) straw phantom 1: initial position, (d) straw phantom 1: 90° rotated, (e) straw phantom 2: initial position, (f) straw phantom 2: 90° rotated.

In the conventional single-transmit channel MR scanner, the circularly polarized magnetic field, *H* ^+^, can be measured by *B*1 mapping methods, but the other two components are hard to measure. If only one measurement of magnetic fields, *H* ^+^ is available, the anisotropic information is lost in the estimate of the circulating admittivity. Even if only one component of the magnetic fields, *H* ^+^, can be measured, the anisotropic information can be acquired by measuring several *H* ^+^ by rotating the object with respect to transmit coil. In Figures 10 and 11, the circulating conductivities only with *H* ^+^ for two positions of the object, that is, initial position and 90° rotated, are shown. For simulation data shown in Figure 10, the circulating conductivities were computed with both magnitude and phase of *H* ^+^ or with only phase of *H* ^+^. For experimental data shown in Figure 11, only the phase of the *H* ^+^ was used. For isotropic tissues, circulating conductivity was not related to the position, but for anisotropic tissues, the circulating conductivity at the center of the phantom was changed. At the boundary of the phantom, very high or negative, especially at the top and the bottom of the phantom after rotating 90°, conductivity values were observed. We think that boundary artifacts [16] created at the air-water boundary were spread inside the phantom due to the spatial filtering used to reduce the noise in the conductivity estimates and thus our conductivity estimates at the boundary of the phantom were not reliable.

## 5. Conclusions

 Noninvasive measurement of conductivity tensor at Larmor frequency could be achieved using MRI. Using measured *B*1 maps from MRI, circulating current density and circulating electric fields can be estimated. In this work, the ratio of the two, called circulating admittivity, was proposed as measure of the conductivity anisotropy at Larmor frequency. Given eigenvectors of the conductivity tensor, quantitative measurement of the eigenvalues can be achieved from circulating admittivity for special tissue models. Without eigenvectors, qualitative information of anisotropy still can be acquired from the distribution of the circulating admittivity. The limitation of the circulating admittivity is that the anisotropic information is lost if only one component of the magnetic field is available. At least, an additional acquisition, either by rotating the object or some other scheme, needs to be performed for anisotropic information.
